# Molecular mechanisms associated with the strength of the anti-CMV response in nonagenarians

**DOI:** 10.1186/1742-4933-11-2

**Published:** 2014-01-31

**Authors:** Saara Marttila, Juulia Jylhävä, Laura Kananen, Antti Hervonen, Marja Jylhä, Mikko Hurme

**Affiliations:** 1 Department of Microbiology and Immunology, School of Medicine, University of Tampere, Tampere, Finland; 2 Gerontology Research Center, Tampere, Finland; 3 School of Health Sciences, University of Tampere, Tampere, Finland; 4 Center for Laboratory Medicine, Tampere University Hospital, Tampere, Finland

**Keywords:** Cytomegalovirus, Anti-CMV IgG titre, Nonagenarians, Elderly, Transcriptomics, Immune response, Pathway analysis, Upstream regulator

## Abstract

**Background:**

Infection with human cytomegalovirus (CMV) affects the function and composition of the immune system during ageing. In addition to the presence of the pathogen, the strength of the immune response, as measured by the anti-CMV IgG titre, has a significant effect on age-related pathogenesis. High anti-CMV IgG titres have been associated with increased mortality and functional impairment in the elderly. In this study, we were interested in identifying the molecular mechanisms that are associated with the strength of the anti-CMV response by examining the gene expression profiles that are associated with the level of the anti-CMV IgG titre.

**Results:**

The level of the anti-CMV IgG titre is associated with the expression level of 663 transcripts in nonagenarians. These transcripts and their corresponding pathways are, for the most part, associated with metabolic functions, cell development and proliferation and other basic cellular functions. However, no prominent associations with the immune system were found, and no associated transcripts were found in young controls.

**Conclusions:**

The lack of defence pathways associated with the strength of the anti-CMV response can indicate that the compromised immune system can no longer defend itself against the CMV infection. Our data imply that the association between high anti-CMV IgG titres and increased mortality and frailty is mediated by basic cellular processes.

## Background

Cytomegalovirus (CMV), a common herpes virus also known as HHV5, initially causes an asymptomatic primary infection and then remains latent in the body, typically in the myeloid cell compartment. Primary CMV infection typically occurs in childhood, and the seroprevalence in the population increases with age, reaching 85-90% at ages of 75-80 years [1].

Strong evidence suggests that CMV-seropositivity is associated with dramatic changes in the immune system, especially in the T cell compartment. In the CD4+ and CD8+ T-cell populations, the proportion of naïve cells is decreased, while the proportion of memory cells is increased in CMV-seropositive individuals [2-5]. However, the specific role of CMV in these ageing-associated changes remains controversial, although it can be said that CMV at least accelerates detrimental changes [1]. In a recent transcriptomic analysis, we have shown that the CMV-associated and CMV-independent ageing effects on the immune system are different: CMV seropositive nonagenarians show alterations in dendritic cell maturation, B cell development and T helper cell differentiation that are not present in CMV seronegative nonagenarians [6].

Several epidemiological studies imply that the strength of the anti-CMV response, quantitated based on the anti-CMV titre, is of significance in age-related pathogenesis (reviewed in [1]), and high anti-CMV titres are associated with all-cause and cardiovascular mortality [7-10]. Our previous results show that CMV infection and anti-CMV titre are also associated with impaired vascular function in males at a young age [11]. A high anti-CMV titre has also been linked with increased frailty and functional impairment [12-14], although conflicting reports exist with regard to this association [15]. The anti-CMV titre has also been shown to correlate with the T cell proportions [16].

Several factors make the analysis of the exact role of CMV in the pathogenesis of these changes in the human immune system difficult. As the primary CMV infection is typically asymptomatic, CMV resides in cells in a latent form with the potential to reactivate, and reinfection with a new strain is also possible, a study that includes a long follow-up period would be required to obtain information about the effects of reactivation and reinfection on the level of anti-CMV IgG titres and to further understand the significance of these processes on the phenotypic changes that are observed in the elderly. In addition, the direct measurement of the anti-CMV immune response (i.e., the T cell response or antibody formation against the various CMV epitopes) is challenging, and these methods are rarely available in a long-term follow-up setting.

To obtain detailed information on the molecular mechanisms that are associated with the strength of the anti-CMV immune response, we performed a genome-wide transcriptomic analysis of peripheral blood mononuclear cells (PBMC) from 138 CMV-seropositive nonagenarians and have correlated the transcript data with anti-CMV IgG titres.

## Results

Our analysis was performed using the 2010 cohort of the Vitality 90+ study population. This study cohort consists of individuals born in 1920 and living in the city of Tampere, Finland. Home-dwelling and institutionalised individuals are included in this study cohort, and all are of Western European descent. The incidence of CMV seropositivity was high, as 96% (138 of 144) of individuals were anti-CMV IgG seropositive. As in previous studies (see Background), a higher titre was associated with functional impairments in nonagenarians as the anti-CMV IgG titre was inversely correlated with handgrip strength (n = 130, Spearman’s rho = -0.233 p = 0.011) and the Barthel index (n = 138, Spearman’s rho = -0.218, p = 0.010).

To elucidate the molecular mechanisms that are associated with the strength of the anti-CMV immune response, as measured by the anti-CMV IgG titre, we performed a correlation analysis between the level of the anti-CMV IgG titre and global gene expression levels. In the correlation analysis, we found 663 transcripts whose expression level correlated with the level of the anti-CMV titre (Pearson correlation coefficient, Benjamini-Hochberg FDR < 0.20). Of the correlating transcripts, the expression level of 290 transcripts was inversely correlated with the anti-CMV IgG titre, and the expression level of 373 transcripts was directly correlated. The transcripts with the highest correlation coefficients were EIF4BP3 (Pearson’s r = -0.327), LSM4 (r = -0.311), NUAK2 (r = -0.302), TCP1 (r = 0.351), KIAA0947 (r = 0.348) and WAC (r = 0.348). A complete list of the correlating transcripts can be found in Additional file 1: Table S1.

**Figure 1:**
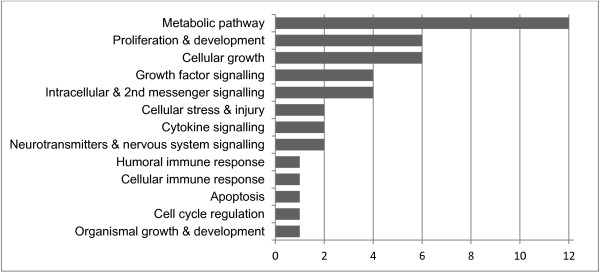
**Pathway categories that were affected by the level of the anti-CMV IgG titre.** The numbers of pathways in the indicated pathway categories for nonagenarians are listed. Canonical pathways that were affected by the anti-CMV IgG titre are grouped into pathway categories that are defined in the Ingenuity Knowledge Base^®^. One canonical pathway can belong to several categories.

We then performed canonical pathway analysis to identify the pathways that are affected by the level of the anti-CMV IgG titre. We identified 26 such pathways, and they are listed in Additional file 2: Table S2. The most significant pathways included *3-phosphoinositide biosynthesis*, *Dolichyl-diphosphooligosaccharide biosynthesis* and *3-phosphoinositide degradation*. The canonical pathways were further categorised into pathway categories (Figure 1). The eight most affected pathways were metabolic pathways, and 12 of the 26 affected pathways were metabolic pathways. The most represented signalling pathway categories were *Cellular growth* and *Proliferation and development*.

Upstream regulator analysis was also used to identify molecules that were predicted to be activated or inactivated based on the expression data (Table 1). By definition, the upstream regulators are molecules that can affect the expression of other molecules. In nonagenarians, we identified 4 affected upstream regulators, CD24, Mek, IFNL1 and AR, all of which were predicted to be activated.

**Table 1:** Upstream regulators associated with the level of anti-CMV IgG titre

**Upstream regulator**	**Predicted activation state**	**Activation z-score**	**Target molecules in dataset**
CD24	Activated	2.449	ADD3,EDEM3,MBNL1,SMC4,SPG11,SYNE2
Mek	Activated	2.446	ABCE1,DUSP6,LYAR,MOB4,NOP58,OSBPL8
IFNL1	Activated	2.000	GBP1,SAMD9,STAT1,TDRD7
AR	Activated	2.000	ACTR3,CAST,CTSO,STAT1,WIPF1

Upstream regulators in nonagenarians that are predicted to be associated with the anti-CMV IgG titre are listed. An activation z-score >2 or < -2 is considered significant. Target molecules are the molecules in our data set on whose expression the prediction about the upstream regulator is based.

We also aimed to repeat the correlation analysis in young controls (n = 30). The incidence of anti-CMV seropositivity was lower in the young controls compared with nonagenarians as 63% (19 of 30) of the controls were anti-CMV IgG seropositive. Additionally, the median CMV titre was significantly lower in young controls compared with nonagenarians (7 200 and 19 500, respectively, Mann–Whitney U-test, p < 0.001). Details of the anti-CMV IgG titres are presented in Additional file 3: Table S3. However, we found no transcripts that were significantly associated with the level of anti-CMV IgG titre in the young controls (Pearson correlation coefficient, Benjamini-Hochberg FDR < 0.20).

## Discussion

The aim of this analysis was to identify the molecular mechanisms that are associated with the strength of the anti-CMV response and to thus shed light on the association between high anti-CMV IgG titres and increased mortality, frailty and functional impairment in the elderly. We identified 663 transcripts that were associated with the strength of the anti-CMV response and further characterised these using pathway analysis tools. The data indicate that the strength of the anti-CMV response in nonagenarians has widespread effects on the cells of the immune system that range from basic cellular processes to metabolic processes and immune functions.

The majority of the canonical pathways that were associated with the level of anti-CMV IgG titre were metabolic pathways, e.g., the cholesterol and inositol processing pathways. Of the remaining pathways, classified as signalling pathways, the most represented categories were *Cellular growth* and *Proliferation and development*. Only two immune system-associated pathways were represented, and interestingly, none were explicitly associated with B cell function. Based on the pathway analysis, it appears that the effects of the strength of the anti-CMV response are focused on the basic functions of the cell in nonagenarians.

Additionally, when studying the single transcripts that were associated with the level of the anti-CMV IgG titre, it can be observed that the basic functions of the cell are represented. The top 20 correlated transcripts (directly and inversely) included several translation and mRNA processing-related genes, ER-associated genes and different signalling molecules. When studying the groups of directly and inversely correlated transcripts, transcripts associated with metabolic processes, signal transduction, gene expression and RNA processing, as well as cell death, could be found in both groups. For a list of all transcripts that were associated with the anti-CMV-IgG titre, see Additional file 1: Table S1.

However, when studying single transcripts or canonical pathways, the direction of change in the different cellular processes cannot be identified. The analysis only indicates the affected pathways based on the transcripts that correlate with the anti-CMV titre. Therefore, the activation or inhibition status of a given pathway cannot be directly evaluated.

To gain information about the direction of changes that are associated with an increased anti-CMV response, we used the upstream regulator analysis tool. This analysis can identify the molecules that may be responsible for the gene expression changes that were observed in the data. In our analysis, all identified upstream regulators were predicted to be activated.

CD24 has not previously been associated with CMV infection. However, polymorphisms in this gene are known to affect the outcome of hepatitis B infection [17] and possibly other viral infections. Interestingly, homology exists between CD24 and the CMV UL139 ORF [18].

Mek is a complex that consists of MAP2K1-7. This group of proteins has not been directly linked to CMV. However, they are a part of the mitogen-activated phosphorylation cascade that mediates a wide range of cellular signals that regulate processes such as cell growth, differentiation, survival and immune responses [19].

The activation of the androgen receptor (AR) in nonagenarians is unexpected. To our knowledge, the effect of androgens on CMV infection has been studied only in prostate cancer cells [20]. These investigators showed that androgen activates the CMV major immediate early (MIE) promoter via a protein kinase A-mediated pathway. Thus, the androgen-dependent mechanism that is involved in the up-regulation of the anti-CMV immune response remains unknown. However, it is possible that the association between CMV responses and androgens is indirect; for example, androgens may enhance CMV-induced cell damage, thereby increasing the release of immunogenic viral antigens. Our recent finding demonstrating that the association between high anti-CMV titres and cardiovascular changes is restricted to males [11] may support this explanation.

IFNL1 is a type III interferon that is known to have similar antiviral properties as group I interferons. However, the magnitudes of gene expression changes and antiproliferative effects between type I and type III interferons are different [21,22]. Nearly all cell types express IFNLs after viral infection, and it can be assumed that most viruses can induce their expression [21]. In mice, IFNL1 has been shown to decrease the number of hCMV immediate-early 1 protein-positive cells *in vitro*[23]. The predicted activation of IFNL1 was the only viral defence-associated feature that was linked with the strength of the anti-CMV titre in our study. Because the immune systems of elderly individuals are compromised, this defence mechanism may offer an alternative solution in an attempt to control the CMV infection.

However, the lack of a strong association with viral defence may help to explain the adverse effects that are associated with high anti-CMV IgG titre. Upon possible CMV reactivation, the compromised immune system of an elderly person is unable to produce the necessary anti-viral reaction, thus leading to adverse outcomes for the individual.

In our analysis, the anti-CMV IgG titre was associated with few viral defence processes, and the majority of associations were with the basic cellular processes. Our results imply that the detrimental effects of high anti-CMV IgG titre are mediated by changes in basic cellular processes. Unfortunately, the direction of change in the cellular processes that are associated with the anti-CMV IgG titre cannot be deduced from our data. However, in many analyses where the association of high anti-CMV IgG titre and mortality or frailty have been studied, other confounding factors have been taken into account [7-10,12]. Thus, it can be speculated that the effect of high anti-CMV titre is not merely a bystander effect, i.e., the condition of the individual deteriorates for one reason or another, thus leading to increased frailty and mortality and elevated anti-CMV IgG titre, but that it can, in some cases, also have a causative effect on the adverse outcomes.

In the young controls, we could not identify any transcripts whose expressions were associated with the level of the anti-CMV IgG titre. This finding may be due to inadequate statistical power because we only had 19 CMV seropositive young controls. However, the result may also reflect a true biological situation, i.e., the effects of CMV are less pronounced in young controls. It is also possible that the immune systems of young individuals have been in contact with the virus for a shorter period of time compared with those of nonagenarians, and thus, the associations may not be identifiable by our methods.

## Conclusions

This study identified the molecular mechanisms that are associated with the strength of the anti-CMV response. The majority of the affected processes were those of basic cellular functions, and no notable associations with immune system pathways were observed. The reported associations between high anti-CMV IgG titre and mortality and frailty are most likely mediated by these basic cellular processes.

## Materials and methods

### Study population

The study population consisted of 144 nonagenarians, 138 of which were CMV seropositive (CMV+), and 30 young controls (19-30 years of age, median 23 years), of which 19 were CMV seropositive. All of the study subjects were of Western European descent. The nonagenarians were a part of the on-going Vitality 90+ study, a prospective study involving home-dwelling and institutionalised individuals who were 90 years of age and lived in the city of Tampere. The nonagenarians who took part in this study were recruited as described previously [24]. The study protocol was approved by the ethics committee of the city of Tampere. The young controls were healthy, non-smoking laboratory personnel who lacked any medically diagnosed chronic diseases or infectious conditions two weeks prior to blood sample drawing.

### Sample collection

The blood samples were collected between 8 and 12 am by a home-visiting, trained medical student. The samples were collected into EDTA-containing tubes and were directly subjected to PBMC separation using Ficoll-Paque density gradients (Ficoll-Paque™ Premium, cat. no. 17-5442-03, GE Healthcare Bio-Sciences AB, Uppsala, Sweden). The PBMC layer was collected, and the cells were suspended in 150 μl of RNAlater solution (Ambion Inc., Austin, TX, USA) for use in microarray analyses. The plasma layer was collected and stored at -70°C.

### CMV serology

The anti-CMV IgG titre was measured using a commercial enzyme-linked immunosorbent assay kit (Enzygnost^®^ Anti-CMV/IgG, Siemens Healthcare Diagnostics Products GmbH, Marburg, Germany) to test the stored (-70°C) plasma. The analysis was performed in the laboratory of Tampere University Central Hospital, which is accredited according to international standards (EN ISO/IEC 17025). Seropositivity for CMV was defined according to the manufacturer’s instructions as a plasma anti-CMV IgG titre ≥ 230.

### Physical performance

Maximal isometric handgrip strength and the Barthel index were measured as described previously [25]. Briefly, handgrip strength was measured using a hand-held dynamometer. Each participant conducted three maximal trials using both hands, and the highest value for the primarily used hand was accepted as the result. The 10-item Barthel index measures the individual’s ability to perform the activities that are required for daily living, such as mobility, transfer, feeding and maintaining personal hygiene. Each of the ten tasks is scored from 0-10, resulting in a total score of 0-100, where 0 reflects total dependence and 100 reflects total independence.

### RNA extraction and microarray analysis

RNA was purified using a miRNAeasy mini kit (Qiagen, Hilden, Germany) according to the manufacturer’s protocol with on-column DNase digestion (AppliChem GmbH, Darmstadt, Germany). The RNA concentration was determined based on the A260/A280 absorption ratio (NanoDrop^®^ ND-1000; NanoDrop Technologies, Wilmington, DE, USA) and using the Agilent RNA 6000 Nano Kit with an Agilent 2100 Bioanalyser (Agilent Technologies, Santa Clara, CA, USA).

RNA (330 ng) was used to prepare labelled cRNA using the Illumina TotalPrep RNA Amplification Kit (Ambion Inc., TX, USA) with overnight incubation according to the manufacturer’s protocol. The quality of the labelled cRNA was controlled using a 2100 Bioanalyser (Agilent).

Labelled cRNA (1 500 ng) was hybridised on a HumanHT-12 v4 Expression BeadChip (Cat no. BD-103-0204, Illumina Inc., CA, USA) according to Illumina’s protocol, and the chips were scanned using Beadscan (Illumina Inc.). The microarray analysis was performed at the Core Facility of the Department of Biotechnology, University of Tartu. The microarray data are available in the GEO database (http://www.ncbi.nlm.nih.gov/geo/) under accession number GSE40366.

### Data processing and statistical analysis

The preprocessing, filtering and microarray data analyses were performed using the Chipster v2.6 program [26] (IT Center for Science Ltd (CSC), Espoo, Finland). The lumi pipeline was used for data preprocessing and normalisation. Array_Address_ID was used as a probe identifier, background correction was performed using the bgAdjust.affy package, and the data were log2-transformed and normalised using the rsn method. The quality of the expression data was verified using a box plot, density plot and principal component analyses (PCA).

To filter out the non-expressed probes and poor quality data, we included only probes with log2-transformed expression values between 5 and 100 on all chips. After applying this cut-off, 10 703 and 8 745 probes remained for statistical testing in the young controls and nonagenarians, respectively. To identify the transcripts with expression levels that correlated with the anti-CMV titre, the anti-CMV titre was log2-transformed and correlated with the expression values using Pearson’s rho and Chipster’s correlate with the phenodata tool. We considered probes with FDR < 0.20 as significant (corresponding to Pearson’s rho above 0.2036 in nonagenarians).

### Pathway and upstream regulator analysis

To identify canonical pathways and upstream regulators that were associated with anti-CMV IgG titres, we analysed the acquired gene sets using the IPA software (Ingenuity^®^ Systems, http://www.ingenuity.com). According to the manufacturer, the canonical pathways are well-characterised metabolic and cell signalling pathways that have been curated and hand-drawn by PhD-level scientists. There are 642 canonical pathways in the Ingenuity Knowledge Base. The information used to construct the canonical pathways was derived from journal articles, review articles, textbooks and the KEGG Ligand database. The canonical pathways are directional, and the affected pathways were grouped into categories that were defined by Ingenuity^®^.

According to the manufacturer, the upstream regulators in the IPA analysis are molecules that can affect the expression of other molecules. The Upstream Regulator Analysis is based on the expected causal effects between upstream regulators and targets, and the expected causal effects are derived from the literature compiled in the Ingenuity^®^ Knowledge Base. The expression value of the upstream regulator itself is not taken into account in the analysis.

All of the data sources provided by the Ingenuity Knowledge Base were included in the IPA analysis. For the association of molecules, only experimentally observed results were accepted, and only human data were considered. The HumanHT-12 v 4.0 was used as a reference set to generate p-values for the pathways, and Fisher’s exact test was used to calculate p-values for the pathways. For upstream regulator analysis, direct and indirect relationships were included.

Using these parameters, 630 analysis ready molecules were found. We considered a canonical pathway to be significantly affected at p < 0.05 and when the pathway contained a minimum of 3 genes. Pathways associated with cancer and other diseases, as defined by Ingenuity Systems^®^, were excluded from the analysis. Because our dataset was not biased (all bias scores were below 0.25), we used a z-score >2 or < -2 as a threshold for a significant upstream regulator. The IPA analysis was performed on the 18th of November 2013.

## Competing interests

The authors declare that they have no competing interests.

## Authors’ contributions

SM performed the experiments, statistical analyses and transcriptomic analyses and wrote the manuscript. JJ performed the experiments and assisted in the statistical analyses, transcriptomic analyses and in writing the manuscript. LK assisted in the statistical analyses. AH and MJ were responsible for recruiting the study population. MH provided the reagents and materials for the study, designed the study and assisted in writing the manuscript. All authors read and approved the final manuscript.

## Supplementary Material

Additional file 1: Table S1 Transcripts correlated with anti-CMV IgG titres in nonagenarians. List of all transcripts whose expression correlates with the level of anti-CMV IgG titres in nonagenarians (Pearson’s correlation coefficient > 0.2036, FDR < 0.20). Click here for file

Additional file 2: Table S2 Canonical pathways that are affected by the level of anti-CMV IgG titre in nonagenarians. P-value from Fisher’s exact t-test. Click here for file

Additional file 3: Table S3 Anti-CMV IgG titers in nonagenarians and young controls. Click here for file
